# Diagnostic value of contrast-enhanced ultrasound combined with serum procalcitonin in tuberculous lymph nodes and metastatic lymph nodes

**DOI:** 10.1016/j.clinsp.2024.100541

**Published:** 2024-12-20

**Authors:** Lin Li, Lan He, Minchao Xiong, Xiaoyan Wang

**Affiliations:** a Department of Radiology, Wuhan Asia General Hospital, Wuhan City, Hubei Province, PR China; b Department of Medical Imaging, Ezhou Central Hospital, Ezhou City, Hubei Province, PR China; c Department of Radiology, The People's Hospital of Zhaoyuan City, Zhaoyuan City, Shandong Province, PR China

**Keywords:** Ultrasonography, Time-Intensity Curve, Procalcitonin, Tuberculous Lymph Nodes, Diagnosis

## Abstract

**Objective:**

To investigate the value of Contrast-Enhanced Ultrasound (CEUS) combined with Procalcitonin (PCT) in differentiating Tuberculous Lymph Nodes (TLN) from Metastatic Lymph Nodes (MLN).

**Methods:**

This prospective cohort study included 207 consecutive patients diagnosed with CTL. Before confirming through pathology or laboratory tests, every patient received standard ultrasound, CEUS, and MRI examinations, with the imaging results analyzed afterward. Serum indicators were measured by Enzyme-Linked Immunosorbent Assay (ELISA). Predictive modeling was performed by multifactorial logistic regression. Evaluate the diagnostic and calibration performance of the predictive model by drawing Receiver Operating Characteristic (ROC) curves and calibration curves, and using Area Under the Curve (AUC) and Hosmer-Lemeshow (H-L) tests.

**Results:**

The presence of lymph node lesions was confirmed by routine ultrasound and MRI in 207 patients, of which 102 (49.27 %) had a pathological or laboratory diagnosis of metastatic lymph nodes (MLN), and 50.8 % were tuberculous lymph nodes (TLN). According to imaging findings of CEUS, TLN was more commonly associated with enhanced concentric performance in the arterial phase (67.65 % vs. 40.95 %) and heterogeneous enhancement pattern in lymph nodes (70.59 % vs. 52.38 %). Peak Intensity (PI) of lesions was higher in patients with MLN. Increased age-enhanced concentric performance in the arterial phase, increased PI, and serum PCT greater than 5.39 ng/mL were independent risk factors for MLN. The prediction model of serum PCT combined with CEUS had a higher diagnostic value for MLN. The H-L test indicated a satisfactory model fit (all p > 0.05), and the calibration curve closely approximates the ideal diagonal.

**Conclusion:**

CEUS combined with serum PCT has better clinical application value in the differential diagnosis of TLN and MLN.

## Highlights

• According to imaging findings of CEUS, TLN was associated with enhanced concentric performance in the arterial phase and heterogeneous enhancement pattern in lymph nodes.
• Peak Intensity (PI) of lesions was higher in MLN patients.
• Increased age-enhanced concentric performance in the arterial phase, increased PI, and serum PCT above 5.39 ng/mL were independent risk factors for MLN.
• The prediction model of serum PCT combined with CEUS had a higher diagnostic value for MLN.

## Introduction

As a result of Tuberculous Lymph Nodes (TLN) and Metastatic Lymph Nodes (MLN), lymph node enlargement is very common. Diagnosis and treatment options differ between these conditions^1^. According to the World Health Organization Report, approximately 10 million people will be infected with Tuberculosis (TB) in 2021.^2^ It is estimated that extrapulmonary TB accounts for 10 % of infections worldwide, and it accounts for 9.2 % to 11.2 % percent of active TB in China.^3^ Cervical Tuberculous Lymphadenitis (CTL) is usually caused by Mycobacterium tuberculosis through infection of the mucous membranes in areas such as the oral or nasal cavities, which subsequently spreads along the lymphatic vessels to the lymph nodes.^4^^,^^5^ CTL has the highest incidence of tuberculous lymphadenitis, with a complex clinical presentation, and identification with other lymph node diseases remains challenging.^6^ Therefore, there is an urgent need for methods that can aid in making a rapid and accurate diagnosis. Accurate diagnostic evaluation of abnormal lymph nodes in the neck is important for appropriate treatment. TB and lymph node metastasis are treated differently clinically, which makes it vital to distinguish them accurately for the benefit of patients.

Ultrasonography (US) is the primary imaging technique for superficial lymph node lesions. Routine US can clearly characterize lymph node size, morphology, internal structure, hilum, or blood flow to identify benign and malignant lymph nodes. Tissue biopsy of lymph nodes can also be visualized by ultrasound. However, there are problems due to the overlap of lymph nodes in morphology and blood flow distribution on ultrasound images and the inability to show low flow rates. Therefore, conventional ultrasound diagnosis limits the identification of different lymph node features.

Contrast-Enhanced Ultrasound (CEUS) is a new diagnostic ultrasound technique that efficiently evaluates and displays real-time information about perfusion and microcirculation within normal tissues and lesions. In addition, Time-Intensity Curves (TIC) quantify blood perfusion in lesions and provide additional diagnostic information for lymph node characterization. CEUS has a higher accuracy in diagnosing benign lymph node disease and tumor lymph node metastasis compared to conventional ultrasound. However, there is less data on the use of CEUS for the differential diagnosis of Tuberculous Lymph Nodes (TLN) from Metastatic Lymph Nodes (MLN).

It has been noted that once infected with TB, the body can initiate an anti-TB immune response involving multiple immune cells and inflammatory factors to manage TB.^7^ Tissues other than the thyroid gland can produce Procalcitonin (PCT) in an inflammatory state, which is markedly increased when the body becomes inflamed to stimulate inflammatory infections accordingly.^8^ Cytokine activation of bystander immune cells, such as monocytes and macrophages, secreting IL-6, Monocyte Chemotactic Protein-1 (MCP-1), and soluble CD40L is closely associated with inflammation or lymph node metastasis of head and neck cancer.^9^, ^10^, ^11^

Here, the authors prospectively investigated the levels of serum biomarkers in patients with TLN and MLN, and compared CEUS in differentiating TLN and MLN, in order to provide more data helpful for differential diagnosis.

## Materials and methods

### Patients

This research employed a prospective cohort design in accordance with the STROBE guidelines. This prospective study involved patients with enlarged cervical lymph nodes who presented to Wuhan Asia General Hospital between January 2022 and December 2023.

Inclusion criteria: 1) Underwent routine US and CEUS measurements; 2) Ultrasound-guided Core Needle Biopsy (CNB) or surgical biopsy with clinically confirmed diagnosis by pathological or laboratory tests.

Exclusion criteria: 1) Patients with incomplete information; 2) Patients who had been treated with anti-tumor therapy before ultrasound examination; 3) Patients with contraindications to CEUS that prevented the examination.

Ultimately, a total of 207 patients aged 21‒72 years (mean age 50.6±16.4 years) were enrolled. Among them 89 were females and 118 were males. The study was approved by the Ethics Committee of Wuhan Asia General Hospital. Informed consent was given by all enrolled patients. According to pathological results, 102 patients had Tuberculous Lymph Nodes (TLN) and 105 patients had Metastatic Lymph Nodes (MLN) (including 21 cases with adenocarcinomas metastases, 25 cases with small cell malignancies metastases, 26 cases with squamous cell carcinoma metastases, and 30 cases with poorly differentiated carcinoma metastases).

### MRI examination

Only the primary scan, i.e. the first MRI, was considered. The MRI examination was performed with a 1.5 T whole-body magnetic resonance imaging (MRI) system (Ingenia 1.5 T; Philips Healthcare, Netherlands). Initially, axial T1-weighted imaging was performed using the FSE sequence with standard receive-only elements encircling the head and neck, followed by axial T2-weighted imaging. Gadopentetate Dimeglumine or gadopentetic acid at 0.1 mmol/kg was injected as contrast agent. Sequences such as T2W-STIR and T2W-FS were used to observe lymph node lesions. It was mainly used to confirm the presence of lesions characterized by cervical lymph nodes.

### US and CEUS examination

A Philips iU-Elite ultrasound system (Washington, D.C., USA) was used, utilizing an L12-5 linear array transducer with a frequency range of 5.0‒12.0 MHz and a mechanical index of 0.06‒0.08. Each patient was placed in the supine position with the neck fully exposed. All cervical lymph nodes were carefully examined for size, shape, internal echoes, and blood flow. Larger lymph nodes were selected, the most typical of which underwent CEUS examination after thorough evaluation. The system was switched to CEUS mode with an L9-3 linear array transducer with a frequency range of 3.0‒9.0 MHz, a mechanical index of 0.13, a power output of 8 %, and a total gain of 27.

Contrast agent, 2.4 mL (SonoVue, Bracco, Italy) was injected intravenously through the elbow vein and then flushed with 10 mL of saline. By optimizing the settings, CEUS was displayed in split-screen mode with the focus located below the lesion to minimize damage to the microbubbles. The entire CEUS examination was dynamically recorded within 3 min and stored. Image acquisition was performed by a standardized trained sonographer, and two senior radiologists analyzed the images in a double-blind manner, including enhancement pattern, homogeneity, degree, perfusion defect, and perfusion mode. A third senior radiologist would review the images and reach a final decision through discussion with the first two radiologists when their analyses were not in agreement.

According to Rubaltelli et al., the contrast agent in the lymph nodes is divided into arterial and parenchymal phases.^12^ The arterial phase^13^ can be divided simply into (1) Centrifugal perfusion: the contrast agent is gradually distributed to the periphery starting from the central hilum (2) Centripetal perfusion: in the contrast agent is gradually distributed from the edge of the lymph node to the center. The parenchymal phase^14^ is divided as follows: (1) Homogeneous enhancement: the entire lymph node shows significant and homogeneous contrast distribution; (2) Heterogeneous enhancement: there are focal areas of low or no enhancement within the parenchyma where the lymph node is markedly enhanced; and (3) Weak enhancement: the entire lymph node is very poorly enhanced, with a uniform or non-uniform distribution of contrast.

All DICOM data were analyzed using the LOGIQ E9 built-in TIC analysis software.^15^ Regions of interest, preferably including intact lymph nodes, were selected to obtain contrast-enhanced TIC and blood perfusion parameters. A cross-section adjacent to the lymph node was used as a control. Quantitative parameters included Peak Intensity (PI), Time-To-Peak intensity (TTP), and Area Under the Curve (AUC, i.e., the area under the curve formed from increasing to decreasing peak intensity at the start of contrast injection).

### Blood sample collection

The sample collection and processing protocol was the same.^16^ The samples were collected in sterile vacuum collection tubes without anticoagulants, left at room temperature for 2 h or overnight at 4°C, and centrifuged at 2000 g for 15 min. The supernatant was collected and stored at -80°C. All serum samples were thawed only once before use.

### Serum biomarker measurements

Enzyme-linked immunosorbent assay was used to measure PCT (Sigma-Aldrich), IL6 (R and D Systems, MN, USA), MCP1, and CD40L (Biosource, Invitrogen).

### Data analysis

Categorical variables were expressed as frequencies (%) and analyzed with and Person's chi-square. For numerical variables, Shapiro-Wilk analysis was used to test the normality of the data. Variables in normal distribution were expressed as mean ± standard deviation, and differences between groups were compared using an independent Student's *t*-test. Skewed distribution variables were expressed as medians (25^th^‒75^th^ percentiles [IQR]) and compared with the Mann-Whitney *U* test. Logistic regression was used to screen for factors associated with MLN. The predictive validity of serum PCT combined with CEUS and TIC characteristics model was assessed using Receiver Operating Characteristic curves (ROC) and calculating AUC, based on the results of the Hosmer-Lemeshow test (p > 0.05), which indicated that the model was well-fitted. R language software package 4.0.5 draws an optimized calibration curve graph, and the Hosmer Lemeshow test (H-L test) evaluates the calibration performance of the model. If the p-value is greater than 0.05, the goodness of fit is good, and the model has good accuracy. SPSS software 22.0 was utilized for analysis and GraphPad Prism 9.5.0 for plotting the images, p-values below 0.05 (2-tailed) were considered statistically significant.

## Results

### Routine ultrasound examination of patients

Among the studied cases, the pathological findings showed that out of 207 patients with enlarged cervical lymph nodes, 102 patients with MLN and 105 with TLN. A greater proportion of TLN patients were female compared to MLN patients (p = 0.002). TLN patients were younger (p < 0.0001). There was no difference in Lymph node Size (L/S) between the two groups (p > 0.05). Most of the patients had well-defined lymph nodes, and a higher proportion of MLN patients had poorly defined lymph nodes compared with TLN patients (Table 1) (p = 0.047). Conventional ultrasound showed no significant differences in internal echo homogeneity, echogenicity, high-level echo, absence of hilum, and blood flow distribution between the two groups (Table 1).

**Table 1:** Clinical characteristics and conventional ultrasound results of patients.

	MLN (n = 102)	TLN (n = 105)	p-value
**Sex, n ( %)**			0.022
Male	50 (49.02)	68 (64.76)	
Female	52 (50.98)	37 (35.24)	
Age, years	42.3 [35.3‒62.3]	38.36 [28.36‒58.3]	<0.0001
**L/S**			0.065
≥ 2	24 (23.53)	37 (35.25)	
< 2	78 (76.47)	68 (64.76)	
**Border**			0.047
Sharp	67 (65.59)	82 (78.10)	
Indistinct	35 (34.31)	23 (21.90)	
**Homogeneity**			0.541
Homogeneous	25 (24.51)	22 (20.95)	
Heterogeneous	77 (75.49)	83 (79.05)	
**Echogenicity**			0.936
Hypoechoic	48 (47.06)	50 (47.62)	
Isoechoic and Hyperechoic	54 (52.94)	55 (52.38)	
**Hilum**			0.147
Present	12 (11.76)	20 (19.05)	
Absent	90 (88.24)	85 (80.95)	
**Hyperechoic islands**			0.06
Present	46 (45.10)	34 (32.38)	
Absent	56 (54.90)	71 (67.72)	
**Vascularity patterns**			0.405
Hilar	9 (8.82)	12 (11.43)	
Peripheral	46 (45.10)	36 (34.29)	
Mixed	40 (39.22)	46 (43.81)	
Avascular	7 (6.86)	11 (10.48)	

MLN, Metastatic Lymph Nodes; TLN, Tuberculous Lymph Nodes. Continuous data were presented as median (interquartile range, IQR) or mean ± SD, and categorical data were presented as n ( %) and compared by Chi-Square test. Mann-Whitney test was used to evaluate the difference between the two groups; p < 0.05 was considered statistically significant.

### US and TIC

The CEUS and TIC characteristics of TLN and MLN are provided in Table 2. Sixty-nine out of 102 TLN (67.65 %) and 43 out of 105 MLN (40.95 %) showed centripetal enhancement in the arterial phase (p = 0.002). In the parenchymal phase, heterogeneous enhancement was predominant in patients with MLN (53.92 %), and this percentage was higher than that in patients with TLN (p < 0.05). There was no statistical difference between the two groups of lymph nodes in terms of whether the enhancement was enlarged or not, or perfusion defects (p < 0.05). The Peak Intensity (PI) of the lesion was higher in MLN patients (p < 0.0001), and TTP and AUC were not statistically different between the two groups (p > 0.05).

**Table 2:** CEUS and TIC characteristics of MLN and TLN patients.

	MLN (n = 102)	TLN (n = 105)	p-value
**Enhancement direction**			0.002
Centrifugal	33 (32.35)	62 (59.05)	
Centripetal	69 (67.65)	43 (40.95)	
**Enhancement type**			0.034
Homogeneous	16 (15.69)	11 (10.48)	
Heterogeneous	55 (53.92)	43 (40.95)	
Rim-like	15 (14.71)	16 (15.24)	
Separated-like	17 (16.67)	35 (38.10)	
**Enlargements of the enhancement ranges**			0.444
Present	58 (47.06)	55 (52.38)	
Absent	44 (52.94)	50 (47.62)	
**Perfusion defect**			0.346
Present	76 (74.51)	84 (80.00)	
Absent	26 (25.49)	21 (20.00)	
**TIC**			
PI (dB)	11.45 ± 4.32	10.25 ± 4.03	<0.0001
TTP (s)	16.65 ± 4.36	16.02 ± 5.21	0.248
AUC (dB × s)	810.35 ± 255.36	792 ± 364.25	0.339

TIC, Time Intensity Curve; PI, Peak Intensity; TTP, Time-To-Peak intensity; AUC, Area Under the Curve.

### Serum PCT, IL-6, MCP1 and CD40L levels

**Fig. 1:**
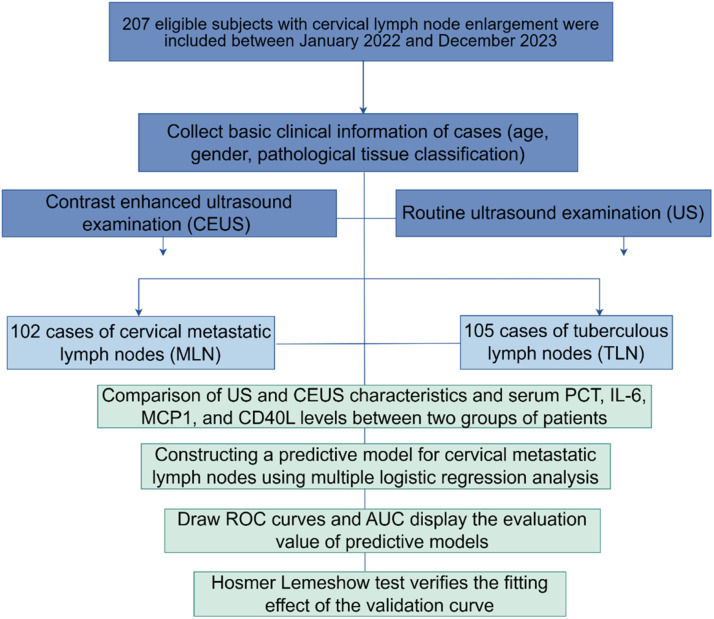
Research flowchart.

Fig. 1 shows the measurements of serum biomarker levels in the two groups of patients. Only serum PCT was observed to be significantly different between the two groups (p < 0.0001), and there were no statistically significant differences in serum IL6, MCP1 and CD40L (p > 0.05). Compared with TLN, higher serum PCT levels were found in MLN patients (p < 0.0001). The authors next categorized serum PCT in all patients into 4 categories based on numerical quartiles (25 %, 75 %, 95 %) as ≤ 2.53 ng/mL (First quartiles), 2.53‒3.58 ng/mL (Second quartiles), 3.58‒5.39 ng/mL (Third quartiles) and ≥ 5.39 ng/mL (Fourth quartiles). Serum PCT was assessed for correlation with MLN.

### Analysis of independent correlates of MLN

The clinical factors, US characteristics, CEUS and TIC characteristics with significant differences based on univariate analysis were included in logistic regression analysis (Table 3). Gender, age, clear border, enhancement direction, enhancement type, PI (dB) and serum PCT quartiles were included. Categorical variables were treated as dummy variables. The final fitting equation is logit (P)=−31.42+6.69×LgAge+1.8×Enhancementdirection+0.36×PI+1.40×PTCquarters(Fourth) Results showed that the risk of developing MLN increased 1.085-fold (95 % CI 1.024‒1.452, p = 0.005) for each additional 10 years of age. The centripetal enhancement in the arterial phase was 1.028 times the centrifugal enhancement (95 % CI 1.006‒1.252, p = 0.014); for each 1 dB increase in PI, there was a 1.034-fold increase in the risk (95 % CI 1.014‒1.054, p = 0.001). Serum PCT greater than 5.39 ng/mL was an independent risk factor for MLN and was 2.58 times greater than in patients with serum < 2.53 ng/mL (95 % CI 1.25‒1.6.36, p = 0.005).

**Table 3:** Multivariate logistic analysis of risk factors for cervical metastatic lymph nodes.

Variables	OR	95 %CI	p-value
Sex			
Female	Reference		
Male	1.525	0.587‒3.251	0.127
Age, years	1.085	1.024‒1.452	0.005
Border			
Sharp	Reference		
Indistinct	1.005	0.358‒1.349	0.425
Enhancement direction			
Centrifugal	Reference		
Centripetal	1.028	1.006‒1.253	0.014
Enhancement type			
Others	Reference		
Homogeneous	0.981	0.758‒1.582	0.425
Separated-like	0.825	0.555‒1.245	0.102
PI (dB)	1.034	1.014‒1.054	0.001
PCT quartiles			
First	Reference		
Second	1.591	0.657‒3.964	0.318
Third	2.09	0.92‒5.32	0.082
Fourth	2.58	1.25‒6.36	0.005

The model incorporates gender confounding factors for correction. OR, Odd Ratio; 95 % CI, 95 % Confidence Interval. p < 0.05 was considered statistically significant.

### Predictive modeling of MLN based on serum PCT, US, and TIC characteristics

**Fig. 2:**
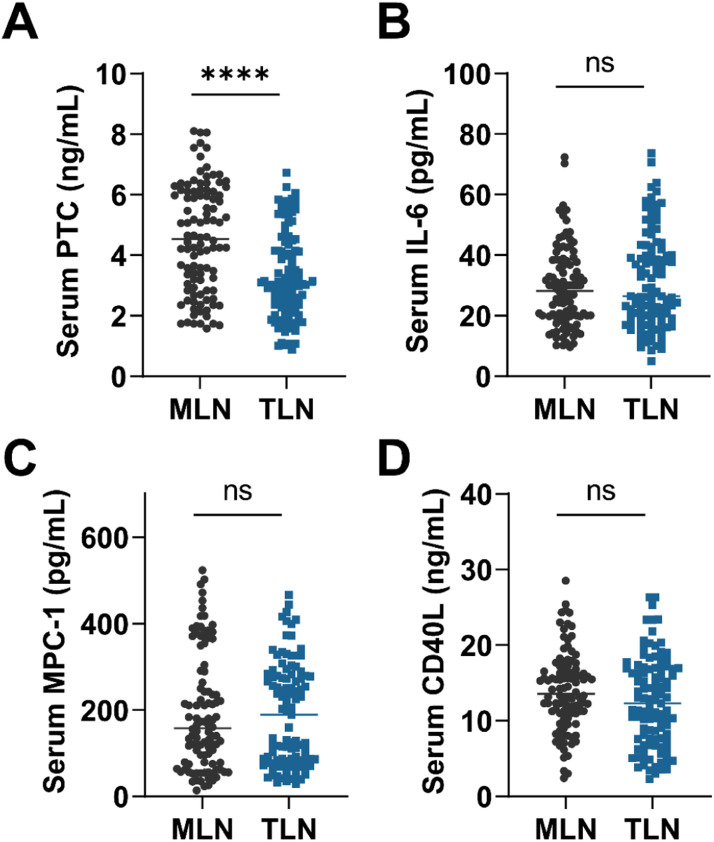
Serum (A) PCT, (B) IL-6, (C) MCP1 and (D) CD40L were compared between MLN and TLN patients. Student *t*-test or Mann-Whitney test was used to assess differences between the two groups. ****p < 0.0001; ns, no statistical difference; p < 0.05 was considered statistically significant.

A prediction model was developed using the independent risk factors associated with MLN obtained from the logistic analysis described above. Predictive values were obtained by binary logistic, followed by ROC analysis. The results were shown by plotting the ROC curve and AUC (Fig. 2, Fig, 3 and Table 4). The serum PCT cut-off value was 4.155 ng/mL, with a sensitivity and specificity of 74.29 % and 59.80 %, respectively. Based on the AUC value, serum PCT was 0.743 (0.652‒0.816), which was higher than the AUC value of model 1 (Enhancement direction and PI) (p < 0.05). The AUC value of model 2 with serum PCT combined with Enhancement direction and PI was 0.763 (0.691‒0.834), which was significantly higher than that of serum PCT and model 1 (p < 0.05). These results suggest that serum PCT has a better differentiation between TLN and MLN and has diagnostic value for MLN. After correcting for age and gender, the model of serum PCT combined with US and TIC characteristics has a higher diagnostic value for MLN. The accuracy of model predictions was assessed through the generation of calibration curves for Model 1 and Model 2 (Fig. 4). H-L test yielded results of 0.085 and 0.107 for Model 1 and Model 2, respectively, both exceeding the threshold of 0.05, suggesting satisfactory accuracy of the models.

**Fig. 3:**
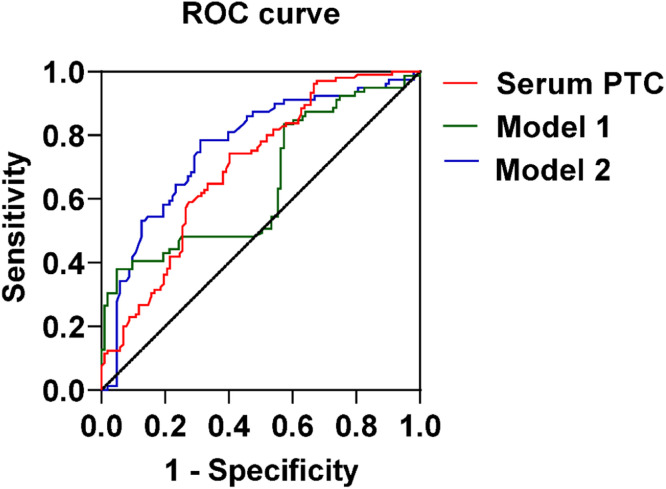
ROC curve of CEUS combined with serum PCT in the diagnosis of TLN and MLN.

**Table 4:** Efficacy of contrast-enhanced ultrasound combined with serum calcitonin in the diagnosis of tuberculous and metastatic lymph nodes.

	Cut off	Sensitivity (%)	Specificity (%)	PPV	NPV	AUC (95 % CI)	p-value
**Serum PCT**	4.155 ng/mL	74.29	59.8	69.32	65.54	0.743 (0.652‒0.816)	<0.0001
**Model 1**	‒	62.36	70.36	46.63	81.36	0.647 (0.565‒0.730)	0.002
**Model 2**	‒	83.86	76.95	73.36	92.56	0.763 (0.691‒0.834)	<0.0001

95 % CI, Confidence Interval; PCT, Procalcitonin; PP, Positive Predictive value; AUC, The Area Under the Curve; NP, Negative Predictive value; Model 1, Independent factors of metastatic lymph nodes in CEUS and TIC characteristics (including Enhancement direction, PI). Age and gender were included as adjustment factors. Model 2, serum PCT combined with Enhancement direction and PI. Age and gender were included as adjustment factors. p < 0.05 was considered statistically significant.

**Fig. 4:**
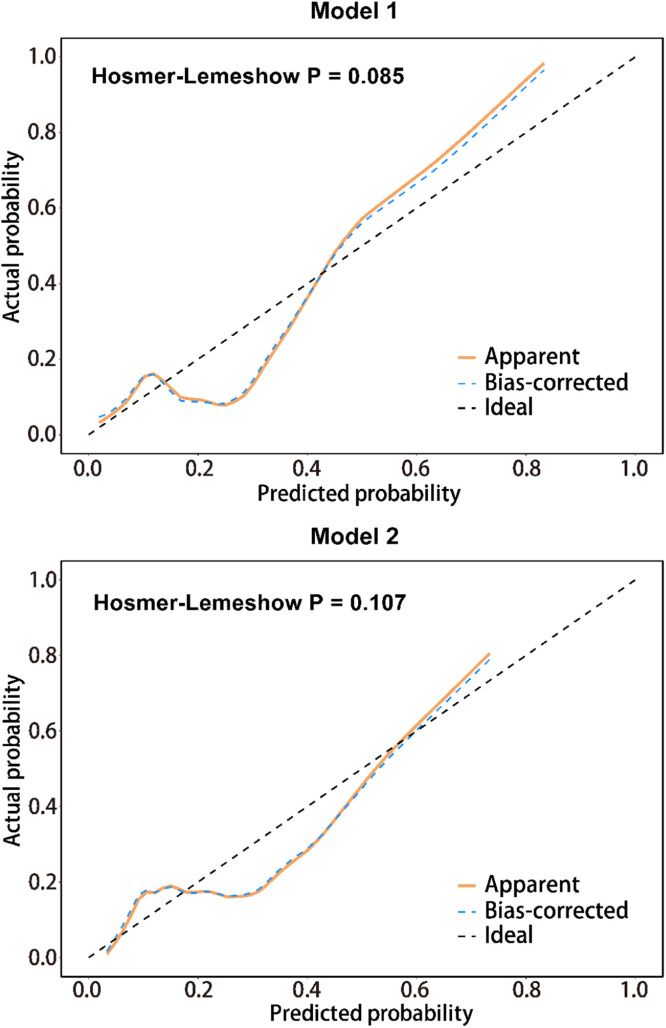
Calibration curve (optimized) and H-L test to evaluate the accuracy of the model.

## Discussion

The overlapping clinical symptoms and imaging features of atypical TLN and MLN make it difficult to perform conventional ultrasound to differentiate between these two lesions.^17^ TLN can exhibit a variety of pathologic changes at different stages of the disease. CEUS improves specificity and sensitivity for benign and malignant lymph nodes with good discriminatory properties.^18^ However, reliance on CEUS alone is still not sufficient for differentiating between these two lesions. In this study, patients were categorized into TLN, and MLN based on the pathological diagnosis. The feature of CEUS presenting a non-centripetal perfusion enhancement pattern and heterogeneous enhancement helped to differentiate between TLN and MLN. The data from the TIC parameter analysis showed higher PI appeared in the patients with MLN. On serum indices, high levels of PCT were observed in MLN. Serum PCT in combination with CEUS and TIC features provided good discrimination between TLN and MLN.

Conventional ultrasound can show the morphology, echo, and internal blood flow of lymph nodes, whereas CEUS can noninvasively assess lymph node microvessel formation, morphological changes, enhancement pattern, and enhancement homogeneity, which can accurately show the state of lymph node microvessel blood flow perfusion. In this study, CEUS features and TIC parameters were analyzed to provide a basis for differential diagnosis. The results of this study showed that conventional ultrasound only observed a higher percentage of unclear borders in patients with MLN compared to patients with TLN (34.31 % vs. 21.90 %, p = 0.047). Binary logistic analysis showed that the lymph node border was not an independent correlate of lymph node metastasis. There were no statistically significant differences in L/S, Homogenicity, Echogenicity, Hilum, Hyperechoic islands, and color Doppler flow imaging. Therefore, it is difficult to differentiate atypical TLN from MLN by conventional ultrasound. Similar reports have also shown that enlarged lymph nodes that may be misdiagnosed as malignant by conventional ultrasound alone are TLN.^19^^,^^20^ In the present study, the authors found more centripetal enhancement in patients with TLN (67.65 %) and slightly more centrifugal enhancement in patients with MLN (49.52 %). Heterogeneous enhancement was more frequent in MLN patients, and the difference between the two groups was statistically significant (p < 0.05), suggesting that enhancement direction and homogeneity in the US can help to differentiate the diagnosis of TLN from MLN.

Peripheral blood vessels are present in tumors with malignant behavior, and tumor growth is controlled by peripheral blood vessels.^21^ The authors speculate that vessel formation may exist in TLN and MLN. Color Doppler Flow Imaging (CDFI) can show blood flow within the tumor and is ineffective in imaging some low-velocity microvessels. Thus, there are drawbacks to showing intranodal vascular features in lymph nodes. Conventional ultrasound and color Doppler ultrasound lack sensitivity in the differential diagnosis of MLN and TLN. In recent years, it has been suggested that microvascular US can help to improve the differential diagnosis of MLN and TLN, but it involves special instruments.^22^ Therefore, the application of this technique is somewhat limited.

In this study, most of the MLN showed heterogeneous enhancement. However, no differences were found in the area of perfusion defects. Although previous studies have shown that when cheesy necrosis of TLN is complete, there is a complete absence of blood supply within the necrotic area, and US demonstrates a complete absence of perfusion within the perfusion-deficient area,^23^ whereas the tumor tissue within the necrotic area of MLN generates tumor vasculature and still has a small amount of blood supply, and therefore a small amount of punctate perfusion remains within the perfusion-deficient area on US.^24^ The authors cannot exclude that the study cohort of patients with TLN were at different clinical stages resulting in inconsistent assessment of perfusion defects. TLN showed more separation-like enhancement than patients with MLN. Separated-like enhancement is associated with a rich blood supply status of the lymph node margins and surrounding areas. In TLN, when cheesy or liquefaction necrosis occurs, this accumulation disrupts the normal vascular structure, resulting in a lack of blood supply to the central lymph node region. There are many granular tissues at the edge of the intact lymph nodes, which are rich in new capillaries. Granuloma formation at the edge of the lymph nodes can cause the immune response of the surrounding soft tissues, resulting in telangiectasia.

Although CEUS parameters can provide detailed diagnostic information, no significant differences in more aspects seem to have been found in this study. Previous studies have shown an increase in the total number of microvesicles and PI values when diseased capillaries open. The tumor metastasizes to the lymph node, which then causes a relative lack of blood flow, thus destroying its normal structure. However, benign lymph nodes do not show this manifestation.^25^ On the other hand, the compression of blood vessels by malignant tumor tissues and the reduction of blood flow in some metastatic lesions lead to lower parameters such as PI, TTP, and AUC in lymph nodes than in benign lymph nodes.^26^ In addition, no significant difference is found in TIC parameters (PI, TTP and AUC) between benign and malignant lymph nodes.^27^ In this study, only PI was observed to be stronger in patients with MLN, with no significant difference in TTP and AUC. The authors believe that this may be influenced by the degree of tissue vasodilatation or other factors such as specific contrast agents, scanner parameter adjustment, and patient metabolism.

Over time, biostatistical techniques have evolved and statistical assessment in biomarker exploration has become more important. PCT, the precursor molecule to calcitonin, is recognized as a systemic inflammatory protein. Since the synthesis and release of PCT is determined by the inflammatory cytokine cascade response during systemic infection, its intensity depends on the number of organisms entering the systemic circulation. It has been previously shown that serum PCT levels are elevated in patients with TB and that serum PCT correlates with the severity of TB in patients.^28^ In addition, it has been reported to be associated with a poor prognosis in TB.^29^ In recent years, serum PCT has been shown to have potential predictive value in diseases associated with lymph node metastasis, especially in head and neck cancer.^30^^,^^31^ Mycobacterium TB is phagocytosed by macrophages and induces cell-mediated immune response and delayed hypersensitivity, macrophage proliferation, and lesion restriction, as well as characteristic tuberculous granulomas and cellular cheesy necrosis, leading to tissue damage. Therefore, this study measured serum PCT and serum IL-6, MCP-1, and soluble CD40L in the cohort of patients. The results showed that serum PCT had higher levels in patients with MLN, while other factors were not statistically different between the two groups. Subsequently, the factors that were statistically different in the US as well as serum PCT were subjected to multifactorial logistic regression analysis, and the results confirmed that the presence of centripetal enhancement in the arterial phase, PI, and serum PCT greater than 5.39 ng/mL were independent risk factors for MLN. The model of serum PCT combined with US and TIC characteristics had a high discriminatory value for MLN.

## Limitations

This manuscript emphasized important CEUS features, but given the potential for inconsistent CEUS features across lymph node classifications, lymph node classifications were not included in this study for further corrective analysis. In addition, based on the half-life of serum PCT, randomized time sampling may have had an impact on the results. Therefore, it is necessary to choose a more standardized detection method to improve the reliability of the data. In future studies, recruiting a sufficient number of patients with different types of lymph node metastases and observing the differences in CEUS characteristics would be beneficial to further observe the differences in CEUS characteristics of lymph nodes in patients with MLN compared with TLN. Follow-up measurement of serum PCT in patients would be beneficial to observe the optimal level of differentiation at the optimal time of measurement to distinguish TLB from MLN.

## Conclusion

CEUS shows real-time perfusion characteristics in lymph nodes. Serum PCT provides a critical value for differentiating TLN from MLN. CEUS combined with serum PCT has clinical value in the differential diagnosis of TLN and MLN.

## Data availability statement

The data that support the findings of this study are available from the corresponding author, upon reasonable request.

## Ethics statement

All procedures performed in this study involving human participants were in accordance with the ethical standards of the institutional and/or national research committee and with the 1964 Helsinki Declaration and its later amendments or comparable ethical standards. All subjects were approved by the Wuhan Asia General Hospital (n° 202101WH-1).

## Funding

Not applicable.

## CRediT authorship contribution statement

**Lin Li:** Conceptualization, Formal analysis, Investigation, Writing – original draft. **Lan He:** Conceptualization, Investigation, Data curation, Writing – original draft. **Minchao Xiong:** Methodology, Formal analysis, Data curation. **Xiaoyan Wang:** Methodology, Data curation, Writing – review & editing.

## Declaration of competing interest

The authors declare no conflicts of interest.
